# Suppressed expression of LDHB promotes age-related hearing loss via aerobic glycolysis

**DOI:** 10.1038/s41419-020-2577-y

**Published:** 2020-05-15

**Authors:** Chunjie Tian, Yeon Ju Kim, Sai Hali, Oak-Sung Choo, Jin-Sol Lee, Seo-Kyung Jung, Youn-Uk Choi, Chan Bae Park, Yun-Hoon Choung

**Affiliations:** 1 Department of Otolaryngology, Dali Bai Autonomous Prefecture People’s Hospital, Dali, Yunnan 671000 China; 2 Department of Otolaryngology, Ajou University School of Medicine, Suwon, 16499 Republic of Korea; 3 Institute for Medical Sciences, Ajou University School of Medicine, Suwon, 16499 Republic of Korea; 4 Department of Medical Sciences, Ajou University Graduate School of Medicine, Suwon, 16499 Republic of Korea; 5 Department of Biomedical Sciences, BK21 Plus Research Center for Biomedical Sciences, Ajou University Graduate School of Medicine, Suwon, 16499 Republic of Korea; 6 Department of Physiology, Ajou University School of Medicine, Suwon, 16499 Republic of Korea

**Keywords:** Translation, Neurological disorders

## Abstract

Age-dependent decrease of mitochondrial energy production and cellular redox imbalance play significant roles in age-related hearing loss (ARHL). Lactate dehydrogenase B (LDHB) is a key glycolytic enzyme that catalyzes the interconversion of pyruvate and lactate. LDH activity and isoenzyme patterns are known to be changed with aging, but the role of LDHB in ARHL has not been studied yet. Here, we found that LDHB knockout mice showed hearing loss at high frequencies, which is the typical feature of ARHL. LDHB knockdown caused downregulation of mitochondrial functions in auditory cell line, University of Bristol/organ of Corti 1 (UB/OC1) with decreased NAD^+^ and increased hypoxia inducing factor-1α. LDHB knockdown also enhanced the death of UB/OC1 cells with ototoxic gentamicin treatment. On the contrary, the induction of LDHB expression caused enhanced mitochondrial functions, including changes in mitochondrial respiratory subunits, mitochondrial membrane potentials, ATP, and the NAD^+^/NADH ratio. Thus, we concluded that suppression of LDHB activity may be closely related with the early onset or progression of ARHL.

## Introduction

Age-related hearing loss (ARHL) that occurs in response to aging is a universal disorder in modern society. ARHL is characterized by an age-associated defect in hearing function, which begins with an increased hearing threshold in the high-frequency region and spreads toward the low-frequency region. Histologically, this process is characterized by the loss of hair cells and spiral ganglion neurons (SGNs) in the cochlea with age^1–3^. The process of ARHL depends on many molecular, physiological, and biochemical changes^4^. Although the exact mechanism remained unclear, ARHL is caused by cumulative effect of extrinsic and intrinsic factors, such as hereditary susceptibility, autophagic stress, inflammation, and oxidative stress^5^.

Decreased mitochondrial function with age has been documented in multiple mammalian species. Studies on mitochondria isolated from human muscle biopsies or rodent muscles support the existence of an intrinsic, aging-dependent mitochondrial defect associated with adenosine triphosphate (ATP) production^6^. Mitochondria are the main source of ATP and a direct intrinsic by-product of this ATP production is reactive oxygen species (ROS). Under normal conditions, ROS are easily scavenged by various endogenous antioxidant enzymes activities, such as superoxide dismutase (MnSOD), catalase (CAT), and glutathione peroxidise (GPX). However, during aging, damaged mitochondria induced less ATP production and aberrant ROS generation, leading to oxidative stress and cellular senescence^7^.

Pyruvate is the substrate used for mitochondrial ATP production and is the product of the final step of glycolysis. In the presence of oxygen, pyruvate is further metabolized in the citric acid cycle to produce NADH and FADH_2_ for oxidative phosphorylation in the mitochondrion^8^. Lactate dehydrogenase (LDH) catalyzes the interconversion of pyruvate and lactate using nicotinamide adenine dinucleotide (NAD^+^) and NADH as a cofactor during the last step of glycolysis. LDH is a homo- or hetero-tetrameric enzyme composed of two subunits, M (muscle) and H (heart), which are encoded by the LDHA and LDHB genes, respectively^9^. Five different isozymes can be formed depending on the ratio of the M and H subunits. The major isozyme in skeletal muscle and liver, with four “M” LDHA subunits, is called LDH5 and mainly contributes to the generation of lactate from pyruvate. Meanwhile, the major isozyme in heart muscles, known as LDH1, contains four heart “H” LDHB subunits and functions in the production of pyruvate from lactate. Isozymes that contain more LDHA subunits will generally undergo reactions to produce lactate from pyruvate, whereas isozymes with more LDHB react to produce pyruvate from lactate. Other variants contain both types of subunits (M3H1:LDH4, M2H2:LDH3, and M1H4:LDH2)^10^. The organ of Corti in the inner ear contains LDH1 (LDHB) rather than LDH5 (LDHA) due to its high-energy requirements^11^.

Conversion from lactate to pyruvate depends on an LDHB-containing isozyme, which is important in maintaining a high level of pyruvate and is highly expressed in tissues with high-energy demands. If the conversion from lactate to pyruvate is disrupted, the substrate concentration for mitochondrial ATP production will decrease, resulting in mitochondrial dysfunction^12^.

In this regard, one study showed that a decreased level of nuclear NAD^+^ contribute to mitochondria defects during skeletal muscle aging in a NAD^+^-dependent deacetylase, sirtuin 1 (SIRT1)-dependent manner. Moreover, calorie restriction slowed aging process by raising NAD^+^ and SIRT1^13^. In addition, augmentation of NAD^+^ by β-lapachone, a known modulator of cellular NAD^+^ by conversion of NADH to NAD^+^, effectively prevented ARHL through the reducing inflammation and oxidative stress, mitochondrial damage in rodents^14^. However, the functional significance of the LDHB in ARHL is unclear. Therefore, the purposes of this study were to elucidate the metabolic changes in ARHL using LDHB knockout (KO) mice and to determine the significance of LDHB in the process of ARHL.

## Results

### Generation of whole-body LDHB KO mice

**Fig. 1:**
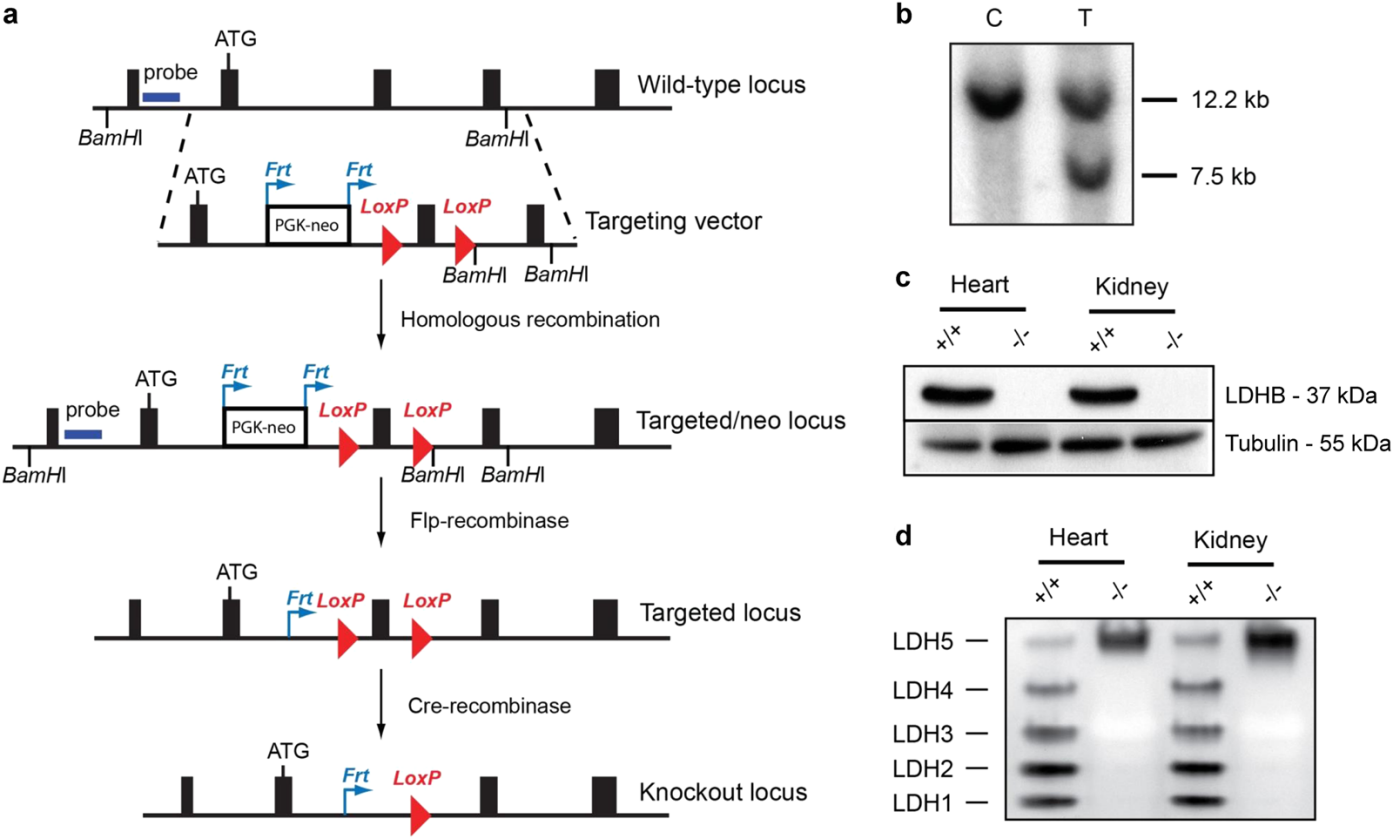
Generation of LDHB KO mice. **a** Targeting of the LDHB gene was performed by floxing exon 3 of the LDHB gene with the PGK-neo cassette containing Frt sites. **b** Screening of target ES cells for LDHB with the probe indicated in Fig. 1a. Control (**c**) and target (T) ES cells. **c**, **d** Confirmation of LDHB knockout in the heart and kidney through western blot analysis (**c**) and gel LDH isomer activity assay (**d**).

To create LDHB gene knockout mouse strains, we first generated LDHB floxed (LDHB loxP/+) mice using homologous recombination in mouse embryonic stem (ES) cells (Fig. 1a). Southern blot analysis revealed ES cells with the target LDHB locus (Fig. 1b). We generated whole-body LDHB gene KO mice by crossing LDHB floxed (LDHB loxP/+) mice with protamine-cre (PRM-cre) mice, which express Cre recombinase in sperm. The resulting double heterozygous mice (LDHB +/loxP, +/PRM-cre) were mated with wild-type (WT) mice to generate heterozygote whole-body LDHB knockout (LDHB +/−) mice, and homozygote LDHB knockout (LDHB −/−) mice were generated by crossing the heterozygote LDHB knockout (LDHB +/−) mice. The generation of whole-body homozygote LDHB (LDHB −/−) mice was confirmed based on complete deficiency of LDHB protein (Fig. 1c) and LDH 1–4 isozymes (Fig. 1d) in the heart and kidney of each KO mouse. In the cochlea, LDHB was broadly expressed throughout the organ of Corti, spiral limbus, spiral ganglion, and spiral ligament, and LDHB KO mice did not contain detectable LDHB protein (Supplementary Fig. 1).

### LDH activity and body weight growth of LDHB KO mice

**Fig. 2:**
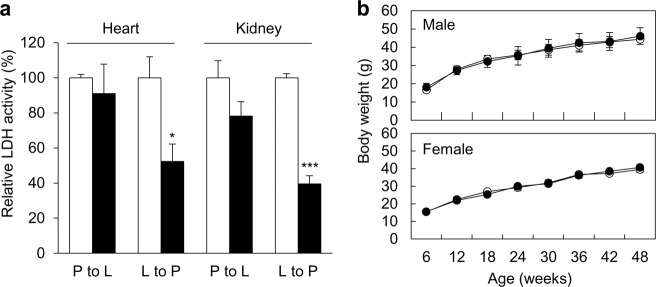
LDH activity and body weight growth of LDHB KO mice. LDH activities decreased in the heart and kidney of LDHB KO mice (**a**). The body weight of LDHB KO mice increased normally (**b**). White bars and circles represent WT mice, and black bars and circles represent KO mice. *P < 0.05, **P < 0.01, ***P < 0.001 vs. WT with Student’s *t*-test.

LDHB is important for maintaining a high level of pyruvate and is highly expressed in tissues with high-energy demands, such as the heart and kidney. In response to KO, LDH activity from the lactate side of the reaction (lactate to pyruvate: L-P) showed a prominent decrease due to deficiency of LDHB in terms of total activity. LDH activity on the pyruvate side of the reaction (pyruvate to lactate: P-L) showed a slight decrease in total LDH activity as well. As the LDHA protein level did not change in LDHB KO mice, total LDH decreased with LDHB deficiency (Fig. 2a). To determine whether loss of the LDHB gene affects the growth of mice, the body weights of male and female groups of LDHB KO mice were measured for 48 weeks. The LDHB KO mice showed normal growth (Fig. 2b).

### Hearing levels and cochlear morphologic findings of LDHB KO mice during aging

**Fig. 3:**
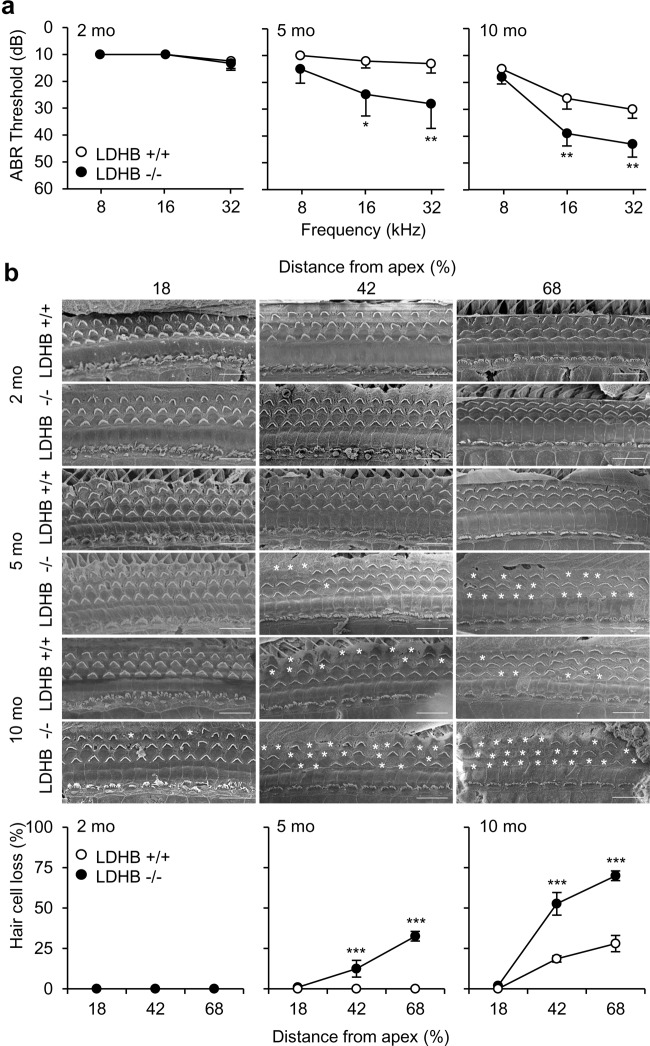
Hearing levels and cochlear morphologic findings of LDHB KO mice during aging. **a** Increased ABR thresholds were observed in LDHB KO mice (−/−, black circle) at the age of 5 months and in WT mice (+/+, white circle) at 10 months. LDHB KO mice showed more severe worsening of hearing than WT mice. **b** SEM findings showed that the number of stereocilia was reduced in the middle (42% distance from apex) and basal turns (68% distance form apex) at 5 and 10 months of age in KO mice and only at 10 months in the WT mice. The difference in hair cell death was statistically significant between the LDHB KO and WT groups of mice. Scale bar = 10 nm. *P < 0.05, **P < 0.01, ***P < 0.001 vs. WT with Student’s *t*-test.

Hearing function was quantified using the auditory brainstem response (ABR) test in WT and LDHB KO mice at 2, 5, and 10 months of age. Both WT and LDHB KO mice showed normal hearing thresholds at 8, 16, and 32 kHz frequencies at the age of 2 months (Fig. 3a). WT mice at 5 months had normal hearing thresholds across frequencies (10.0 ± 0.0 dB/12.0 ± 2.6 dB/13.0 ± 4.2 dB at 8/16/32 kHz, respectively). By contrast, LDHB KO mice exhibited an early onset of progressive hearing loss, with their mean thresholds increasing to 15.0 ± 5.3 dB/24.5 ± 8.0 dB/28.0 ± 9.2 dB at 8/16/32 kHz, respectively. The differences between the two groups were statistically significant at 16 kHz (*P* < 0.05) and 32 kHz (*P* < 0.01) (Fig. 3a). By 10 months of age, ARHL developed at high frequencies in the WT mice, with increased hearing thresholds of 15.0 ± 3.3 dB/26.0 ± 4.0 dB/30.0 ± 3.3 dB at 8/16/32 kHz. The LDHB-KO mice showed worsening of hearing thresholds, reaching 18.0 ± 2.6 dB/39.0 ± 4.6 dB/43.0 ± 4.8 dB at 8/16/32 kHz. Hearing ability decreased for high-frequency sound, while the low-frequency area was preserved. At the age of 10 months, LDHB KO mice showed more severe hearing loss than WT mice. It is widely known that the onset of ARHL begins with high frequencies and spreads toward lower frequency regions during aging^15^. The differences in hearing thresholds between the two groups were statistically significant at 16 kHz (*P* < 0.01) and 32 kHz (*P* < 0.01) (Fig. 3a).

Scanning electron microscopy (SEM) findings showed that the presence of stereocilia on hair cells did not significantly differ between WT and LDHB KO mice at 2 months (Fig. 3b), but the number of stereocilia in the middle and basal turns was reduced at 5 months of age in KO mice. Consistent with ABR results indicating higher hearing thresholds at 32 kHz, samples of high-frequency areas showed fewer remaining hair cells than those at 16 kHz. The difference in the number of hair cells was statistically significant between the two groups both at 16 kHz (*P* < 0.01) and 32 kHz (*P* < 0.01) (Fig. 3b). Hair cell loss was detected in 10-month-old WT mice in the middle and basal turns, but not in the apical turn. By contrast, 10-month-old KO mice had hair cell loss across all turns, including the apical turn. Closer to the base, fewer hair cells remained. The numbers of hair cells in 10-month-old KO mice were lower than those in 10-month-old WT mice in all turns, and statistical significance was detected in the middle and basal turns (*P* < 0.01) (Fig. 3b). Cochlear surface preparation at three different age points (2, 5, and 10 months) across frequencies indicated no inner hair cell loss for either group of animals (Fig. 3b).

### **Cellular change of cochlear lateral walls**

**Fig. 4:**
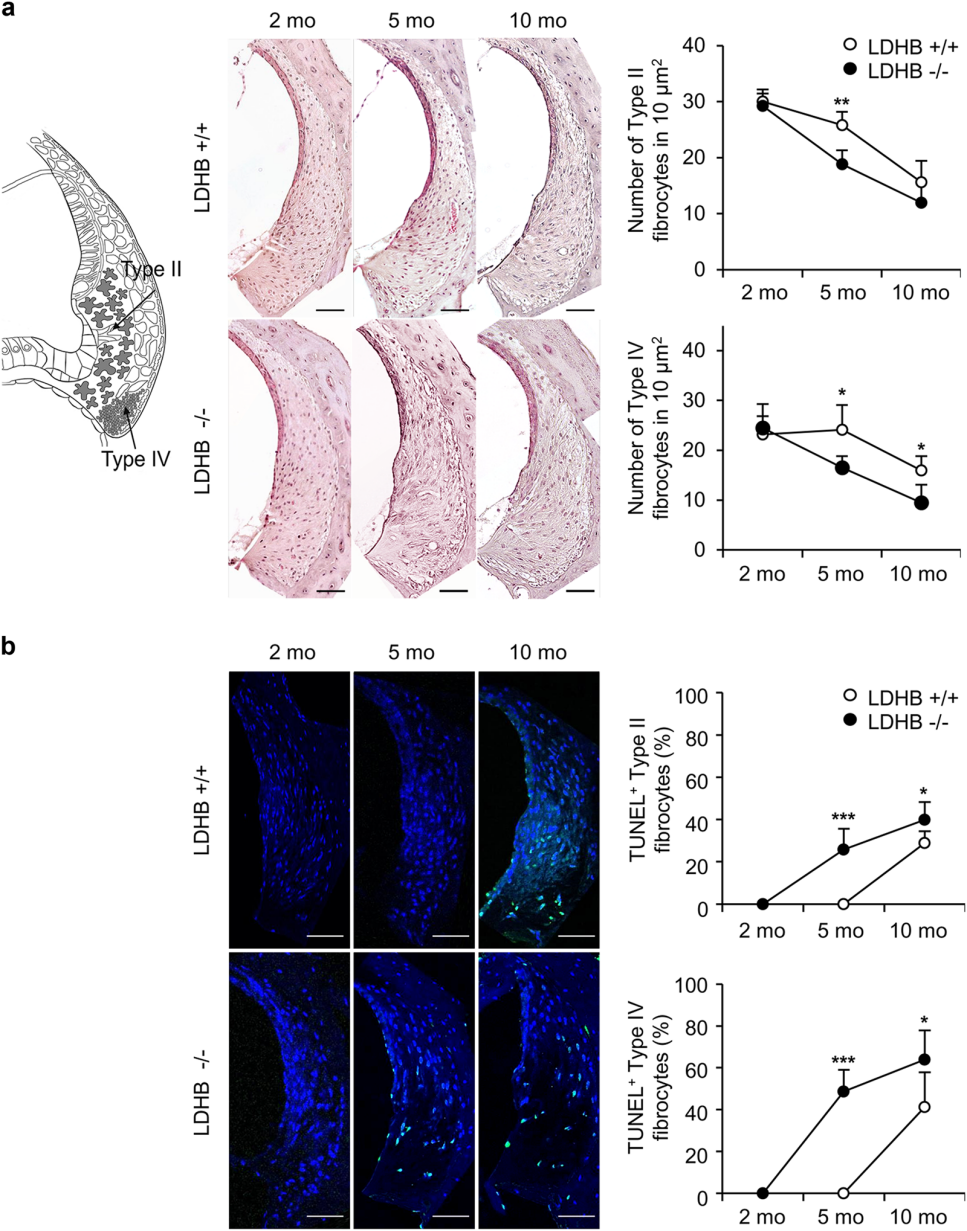
Cellular changes of the cochlear lateral wall. **a** Cell counts of type II and IV fibrocytes in the lateral wall were reduced significantly in LDHB KO mice (−/−, black circle) at 5 and 10 months compared to WT mice (+/+, white circle). **b** Detection of apoptosis with the TUNEL assay in the cochlea of the LDHB KO and WT mice. TUNEL-positive apoptotic cells (green color) were significantly enhanced in the KO mice compared to WT mice. Blue shows DAPI staining of the nucleus. Scale bar = 50 μm. *P < 0.05, **P < 0.01, ***P < 0.001 vs. WT with Student’s *t*-test.

Different types of fibrocytes are shown in a diagram of the lateral wall (Fig. 4a, left). Compared with findings from 2-month-old WT mice, fibrocytes in the lateral wall showed no changes in 5-month-old WT mice, while diffuse degeneration of type II and IV fibrocytes was detected in 5-month-old LDHB KO mice. Comparison of mean cell counts (cells/10.000 μm^2^) in the type II and type IV areas revealed statistically significant differences in both groups at the age of 5 months (Fig. 4a).

The terminal deoxynucleotidyl transferase dUTP nick end labeling (TUNEL) assay was applied to identify cellular apoptosis in the cochlear lateral wall. No differences were observed between 2-month-old WT and KO mice, while TUNEL-positive fibrocytes were detected in 5-month-old KO mice and increased at the age of 10 months. The difference in TUNEL-positive cell expression was significant between the two groups (*P* < 0.01 at 5 months and *P* < 0.05 at 10 months) (Fig. 4b).

### Cellular changes of SGNs

**Fig. 5:**
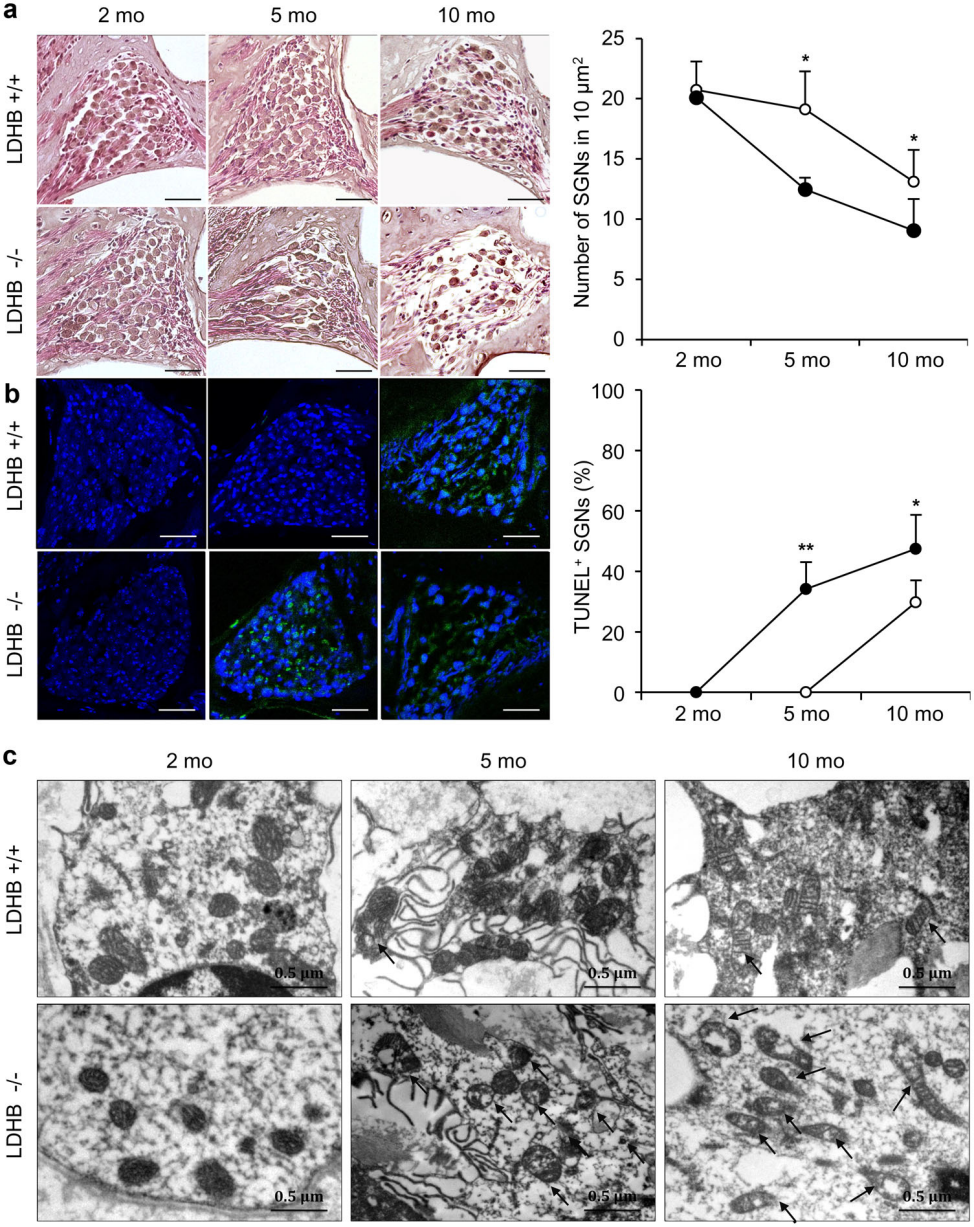
Cellular changes of SGNs. **a** Spiral ganglion cells were significantly reduced in LDHB mice (−/−, black circle) compared to WT mice (+/+, white circle) over time (2, 5, and 10 months). **b** TUNEL-positive cells (green color) were detected much more abundantly in LDHB KO mice than in WT mice at 5 and 10 months. Blue shows DAPI staining of the nucleus. Scale bar = 50 μm. **c** Representative electron microscopy images (20,000×) of cochlear SGNs showed an increased proportion of fragmented mitochondria and distortion of cristae (arrows) in LDHB KO mice compared to WT mice over time. *P < 0.05, **P < 0.01, ***P < 0.001 vs. WT with Student’s *t*-test.

Progressive loss of SGNs through the aging process was detected in the KO mice at the ages of 5 and 10 months, and was observed at 10 months in WT mice. Cell counts (cells/10.000 μm^2^) of SGNs showed statistical significance at the ages of 5 and 10 months (Fig. 5a).

TUNEL-positive spiral ganglion cells were observed in 5-month-old KO mice and increased in 10-month-old KO mice, whereas WT mice showed TUNEL-positive expression only at the age of 10 months, not at 5 months. Differences in the ratio of TUNEL-positive cells to total cells were statistically significant (Fig. 5b).

Considering the essential role of LDHB in mitochondrial function^16^, we evaluated whether there were alterations in mitochondrial morphology in LDHB KO mice through transmission electron microscopy (TEM) analysis. An increased proportion of fragmented mitochondria and distortion of cristae were observed in spiral ganglion cells at the age of 10 months in both WT and KO mice. LDHB KO mice showed more severe mitochondrial fragmentation than WT mice (Fig. 5c).

### LDHB levels and mitochondrial function in differentiated UB/OC1 cells

**Fig. 6:**
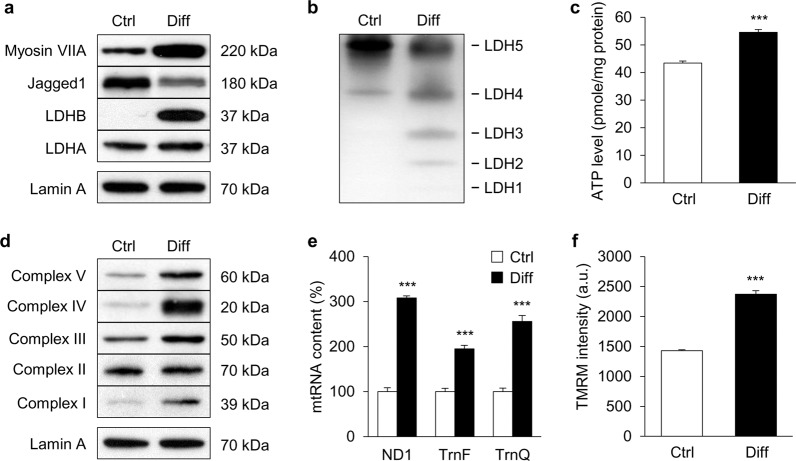
LDHB levels and mitochondrial function of differentiated UB/OC1 cells. UB/OC1 cells were cultured under permissive conditions of 37 °C and 5% CO_2_ without γ-interferon to allow differentiation into hair cells. Expression of LDHB (**a**), ATP level (**c**), mitochondrial respiratory subunits (**d**), mitochondrial RNA levels (**e**), and MMP (**f**) increased in differentiated UB/OC1 cells compared to control cells. **b** The isozyme pattern shifted toward LDH2 in differentiated UB/OC1 cells. White bars represent undifferentiated control cells (Ctrl) and black bars represent differentiated cells (Diff). *P < 0.05, **P < 0.01, ***P < 0.001 vs. undifferentiated cells with Student’s *t*-test.

To investigate the molecular mechanism of hair cell loss in LDHB KO mice in the present study, UB/OC1 cells were used. This cell line can differentiate into hair cells under the conditions of 37 °C and 5% CO_2_^17^. Under differentiation conditions, the hair cell marker Myosin VIIa was upregulated and a supporting cell marker, Jagged 1, was downregulated in differentiated UB/OC1 cells (Fig. 6a). A clear increase of LDHB was observed in differentiated UB/OC1 cells (Fig. 6a, b). By contrast, there was no prominent change in LDHA expression compared with undifferentiated UB/OC1 cells.

The effect of LDHB induction on mitochondrial function was evaluated using two critical indicators of energy status: intracellular ATP and NAD^+^/NADH levels. An increased level of extracellular ATP was observed in differentiated UB/OC1 cells (Fig. 6c). Moreover, increases in the expression of mitochondrial respiratory proteins, mitochondrial RNA content, and mitochondrial membrane potential (MMP) were observed compared to undifferentiated UB/OC1 cells (Fig. 6d, f).

Additionally, NAD^+^/NADH levels, ROS generation, LDH activity, and lactate production were measured (Data not shown). With an increase in mitochondrial respiratory chain activity, the electron donor NADH was significantly converted to NAD^+^, resulting in increases in redox status and NAD^+^/NADH level in differentiated UB/OC1 cells. Mitochondria are known to play a critical role in aerobic ATP generation and are the primary source of ROS for various pathways. We found that intracellular ROS production was upregulated in differentiated UB/OC1 cells. In addition, differentiated UB/OC1 cells had higher LDH activity and accordingly released a larger amount of lactate into the medium than undifferentiated UB/OC1 cells (Supplementary Fig. 2). These results suggest that differentiated UB/OC1 cells upregulated LDHB expression and exhibit increased mitochondrial function.

### **Effect of LDHB knockdown on mitochondrial functions and ototoxic cell death**

**Fig. 7:**
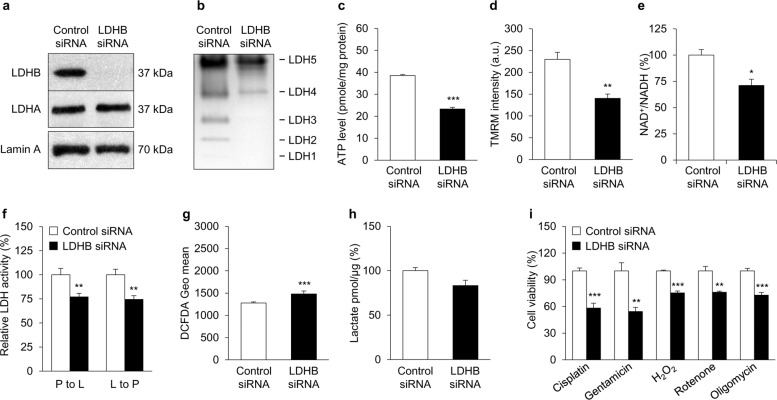
The effect of LDHB knockdown on mitochondrial functions and ototoxic cell death of auditory cells. Expression of LDHA and LDHB (**a**), isozyme patterns of LDH (**b**), ATP levels (**c**), MMP (**d**), NAD^+^/NADH ratios (**e**), and LDH activity (**f**) decreased in UB/OC1 cells treated with LDHB siRNA, while ROS level (**g**) increased compared to the control siRNA cells. **h** The lactate level in culture media was not significantly different between LDHB knockdown and control cells. **i** Quantification of cell viability after cisplatin (2.5 μM), gentamicin (1 mM), H_2_O_2_ (100 μM), rotenone (5 μM), or oligomycin (1 μM) treatment for 24 h. Cell viability in LDHB knockdown cells decreased compared to the control in all conditions. White bars represent control siRNA-treated cells and black bars represent LDHB siRNA-treated cells. *P < 0.05, **P < 0.01, ***P < 0.001 vs. control siRNA-treated cells with Student’s *t*-test.

To investigate the mechanistic relationship between hair cell loss and LDHB, we evaluated mitochondrial functional changes using LDHB small-interfering RNA (siRNA) in differentiated UB/OC1 cells. As shown in Fig. 7a, LDHB protein expression decreased after knockdown, without any change in LDHA expression. The LDH isozyme pattern changed from a mix of five different patterns to favor the LDH5- and LDH4-containing patterns (Fig. 7b). Knockdown of LDHB led to downregulation of ATP production (Fig. 7c), MMP (Fig. 7d), and the NAD^+^/NADH ratio (Fig. 7e). Relative LDH activity decreased in both the P-L and L-P reactions in LDHB siRNA-treated UB/OC1 cells (Fig. 7f). Additionally, LDHB knockdown cells significantly upregulated ROS production compared to control siRNA cells (Fig. 7g). On the other hand, there was no clear change in the amount of lactate released into the medium compared with control siRNA-treated cells (Fig. 7h). Collectively, these results indicate that LDHB knockdown decreased mitochondrial function in differentiated UB/OC1 cells.

Mitochondrial DNA mutations/deletions and excessive ROS production cause mitochondrial dysfunctions, which may be essential mechanisms of age-related auditory disorders^18^. To investigate the sensitivity of LDHB-deficient auditory cells to mitochondrial toxins, a mitochondrial respiratory chain inhibitor, such as rotenone (complex I inhibitor), carbonyl cyanide m-chlorophenyl hydrazone (CCCP; mitochondrial uncoupling agent), antimycin A (complex III inhibitor), or oligomycin (complex V inhibitor) was used. LDHB knockdown significantly increased cell death of UB/OC1 cells compared to control siRNA-treated cells under H_2_O_2_, rotenone, and oligomycin treatments (Fig. 7i). Cisplatin and gentamicin are well-known ototoxic drugs, and auditory cell death is mediated by the generation of ROS^19^. Cisplatin accumulates in mitochondria, causing direct and significant impairment of mitochondrial DNA (mtDNA) and mtRNA^20^. LDHB deficiency significantly increased the sensitivity of UB/OC1 cells to cisplatin and gentamicin (Fig. 7i). These results show that depletion of LDHB in mitochondria makes differentiated auditory cells much more vulnerable to oxidative stress and damage from ROS originating in the mitochondria, and suggest that downregulation of LDHB is a potential contributor to the progression of ARHL.

## Discussion

Mitochondrial pathology plays an important role in different types of hearing loss^21^. Acquired mitochondrial dysfunction in hearing loss, including mtDNA mutation, mitochondrial turnover, and apoptosis, appears to involve increased ROS and decreased energy production, as well as redox imbalance^22^. Tissues that consist of post-mitotic cells, such as brain and inner ear cells, are particularly vulnerable to mitochondrial dysfunction due to their high-energy requirements and inability to undergo regeneration. It is well-known that pyruvate is the substrate of mitochondrial ATP production and final product of glycolysis. LDHB is important for maintaining a high pyruvate level and thus LDHB expression could be directly related to mitochondrial function and energy production. A widely accepted hypothesis for the possible cause of ARHL is mitochondrial dysfunction related to ROS, mtDNA mutation, and ATP deficiency^23^.

In the present study, we generated whole-body LDHB gene KO (LDHB −/−) mice by crossing heterozygous LDHB knockout (LDHB +/−) mice. LDHB KO mice showed a decrease in total LDH activity in both the lactate side of the reaction (lactate to pyruvate: L-P) and the pyruvate side (pyruvate to lactate: P-L). Based on previous research^24^ and our experimental observations, LDHB is highly sensitive to substrate inhibition. At higher concentrations of pyruvate, LDHB activity is inhibited. In our experiments, we selected 0.5 mM pyruvate as the substrate to avoid substrate inhibition. In addition, the LDHA protein level was not affected in LDHB KO mice, and therefore total LDH activity also decreased due to LDHB deficiency on the pyruvate side of the reaction, which is consistent with cell line study results (Fig. 7).

In this study, we have shown for the first time that deficiency of the LDHB gene caused early onset of ARHL using LDHB KO mice. LDHB KO mice showed aggravation of ARHL with an increased number of dysfunctional mitochondria and loss of auditory cells. The hearing threshold shift observed in aging WT mice was similar to a previous description of C57/BL6 mice^4^, whereas LDHB KO mice exhibited more rapid worsening of hearing thresholds at 5 and 10 months of age especially in the high-frequency area, which is characteristic of ARHL. ARHL is mainly rooted in the pathology of cochlear cells, including hair cells, SGNs, and lateral wall fibrocytes. At 5 and 10 months of age, LDHB KO mice displayed earlier and more severe degeneration of hair cells, SGN death, and loss of type II and IV fibrocytes in the lateral wall, which all accompanied by mitochondrial damage. These results indicated that deficiency of LDHB might accelerate degeneration of cochlear cells in the early stage of senescence. However, we cannot exclude the possible contribution of other organs, such as the brain, to the failure of the auditory system in LDHB KO mice. The decline in brain function caused by LDHB deficiency and its impact on ARHL development are under study.

The relationship between LDHB and ARHL is not well-known, but some studies have shown that LDHB activity decreases during the aging process, leading to age-related diseases. Aging WT mice and prematurely aging mtDNA mutator mice had significantly increased LDHA/LDHB activity ratios, which led to elevated lactate levels in the brain^25,26^. Decreased LDHA and LDHB activity has also been demonstrated to be predictive of aging phenotypes in muscle^27^. Regulation of LDH plays a key role in regulating aerobic glycolysis through conversion of pyruvate to lactate through coupled oxidation of NADH to NAD^+^^28^. Recently, decreased intracellular NAD^+^/NADH ratios have been reported in aged tissues. A decrease in the NAD^+^/NADH ratio leads to poly (ADP-ribose) polymerase 1 (PARP1) hyper-activation via altered redox mechanisms and consequent DNA damage^29^. Furthermore, SIRT1 activity and α-ketoglutarate dehydrogenase (α-KGDH) complex, an enzyme complex of the citric acid cycle the ROS generation, are influenced by the NAD^+^/NADH ratio. Recently, PARP1 was identified as an important target in oxidative stress-induced cochlear marginal cell death modulating parthanatos and autophagy^30^. Moreover, some reports have suggested that SIRT1 is abundantly expressed in young cochlear cells and that SIRT1 deficiency may be associated with ARHL^31^. Therefore, the intracellular NAD^+^/NADH ratio may be a critical downstream target in LDHB KO mice, leading to progressive loss of hearing.

We found that an increase of LDHB caused enhancement of mitochondrial function with increases in NAD^+^/NADH balance, ATP level, and mitochondrial respiratory chain subunits observed in differentiated UB/OC1 cells (Fig. 6). The mitochondrial respiratory chain catalyzes NADH oxidation. Increased mitochondrial respiratory chain activity elevates the NAD^+^/NADH ratio and causes translocation of protons across the inner mitochondrial membrane, which ultimately leads to increased ATP production. Knockdown of LDHB caused mitochondrial dysfunction and triggered a decline in the NAD^+^/NADH ratio (Fig. 7). This decrease in the NAD^+^/NADH ratio likely caused mitochondrial dysfunction in UB/OC1 cells.

The increased sensitivity of LDHB knockdown cells to mitochondrial toxins or ototoxic drugs observed in the present study might be caused by mitochondrial dysfunction. Cisplatin and gentamicin are commonly used drugs in the clinic. However, both of these drugs show ototoxicity as a side effect, inducing oxidative stress and mitochondrial defects in the cochlea of the inner ear^19^. The present study showed that LDHB deficiency resulted in mitochondrial defects in auditory cells, which increased sensitivity to ototoxic drugs such as cisplatin and gentamicin.

In conclusion, our findings provide evidence that deletion of LDHB leads to dysfunctional mitochondria, thereby accelerating ARHL. Furthermore, we demonstrated that MMP, ATP, and the NAD^+^/NADH ratio, which are related to mitochondrial function, are reduced in LDHB knockdown cells. These results suggest that LDHB may be used as a useful biomarker for predicting ARHL progression.

## Materials and methods

### Generation of LDHB knockout mouse

The genomic locus of the LDHB gene was modified in mouse ES cells so that two loxP sites flanked exon 3 of the gene. To generate the targeting vector used for genomic modification, we first identified a bacterial artificial chromosome (BAC) clone containing the LDHB gene in a 129Sv RPCI-22M BAC library (Invitrogen) through hybridization with an LDHB cDNA probe. Then, a 10.5-kb genomic fragment containing exons 2–4 of LDHB was excised from the BAC and cloned into pBluescript II SK + (Strategene) through the ET recombination technique to generate a plasmid, pBS-LDHB (Liu, Genome Res). To introduce a loxP site at the intron 3ʹ of exon 3, an oligonucleotide containing the loxP site was inserted at the KpnI site of pBS-LDHB, and the resulting plasmid was designated pBS-LDHBLoxP. Finally, the Frt-PGK-neo-Frt-loxP cassette (Frt-site-flanked neomycin resistance gene with loxP site located at its 3ʹ end) was inserted at the PacI site of pBS-LDHBLoxP to create the LDHB targeting vector, pBS-LDHBKO. The PacI fragment of the Frt-PGK-neo-Frt-loxP cassette was excised from a modified pDELBOY-3X plasmid where PacI sites were introduced at the XhoI and ClaI site (Teglund, Cell). Next, the NotI-linearized LDHB targeting vector was electroporated into ES cells and those clones that grew in the presence of the antibiotic gentamycin were selected. Genomic DNA was isolated from gentamycin-resistant ES cell clones, digested with *Bam*HI, and subjected to Southern blot analysis using a genomic DNA probe located 5ʹ-upstream of the target sequence. Among 130 ES cell clones that were analyzed, two target clones were found. Chimeras were generated through blastocyst injection of target ES cells and germline transmission was achieved from both clones. The Frt-flanked neomycin resistance gene was excised by mating LDHB +/neo-loxP mice to transgenic mice ubiquitously expressing Flp-recombinase, thus generating LDHB +/loxP.

LDHB +/loxP mice were mated with transgenic mice expressing Cre recombinase under the control of the mouse protamine1 promoter (PRM-cre). Prm-cre mice express Cre recombinase during the terminal haploid stages of spermatogenesis. The resulting double heterozygous mice (LDHB+/loxP, +/PRM-cre) were mated with WT C57BL6 mice to generate heterozygote whole-body LDHB knockout (LDHB +/−) mice, and homozygote LDHB knockout (LDHB −/−) mice were generated by crossing heterozygote LDHB knockout (LDHB +/−) mice. All animal procedures were approved by the institutional review board of Ajou University School of Medicine (AJIRB-BMR-SMP-13-181). For all animal experiments, we used five age-matched animals selected randomly from cages of both WT and LDHB KO mice. Animals with abnormal behavior of with obvious rough coat were excluded to minimize individual variance. Researcher who were blinded to the all experimental groups.

### Auditory brainstem response (ABR) analysis

ABR was tested with the Biosig 32 system (Tucker-Davis Technologies, Gainesville, FL, USA), as described previously^31^. Mice were anesthetized with an injection of Zoletil 50 (Virbac Laboratories, Carros, France) and 2% Rompun (Bayer Korea, Ansan, Korea). Prior to placement of earphones, all tympanic membranes were examined with an otoscope. Mice were placed into a sound-shielding booth, which prevented outside noise from disturbing the hearing measurement. The hearing threshold was defined as the lowest intensity at which a clear waveform was visible in the evoked trace and was determined through visual inspection of the responses.

### Morphological evaluation of hair cells using SEM

SEM was used for morphological evaluation of auditory hair cells, as described previously^32^. Three rows of outer hair cells in a given microscopic field were evaluated. Cells were counted in three different microscopic fields of the apical, middle, and basal turns and analyzed statistically^33^.

### **Transmission electron microscopy (TEM)**

Cochleae were dissected and immediately fixed with 2.5% glutaraldehyde in 0.1 M phosphate-buffered saline (PBS), pH 7.4, and maintained at 4 °C for 24 h. Cochlear processing was performed for the TEM study. Cochlear specimens were post-fixed in 1% osmium tetroxide, dehydrated in graded alcohols, and embedded in Spurr’s epoxy resin. Following uranyl acetate and lead citrate staining, specimens were viewed using TEM (EM 902 A; Carl Zeiss AG, Oberkochen, Germany).

### Hematoxylin and eosin staining

Cochleae were fixed with 4% paraformaldehyde in PBS at 4 °C overnight. After decalcification with Calci-Clear Rapid (National Diagnostics, Atlanta, GA, USA) for 2 days and dehydration, samples were fixed in paraffin. Five series of 5-µm sections were obtained using a sliding microtome (Leica, Wetzlar, Germany) in the horizontal plane parallel to the modiolus, after staining with hematoxylin and eosin for histopathology. Fibrocytes in the lateral wall and spiral ganglion cells were examined using light microscopy.

### TUNEL staining

Frozen sections were dried at 37 °C for 1 h. Following permeabilization with 0.2% Triton X-100 and blocking with 1% bovine serum albumin (BSA) for 30 min, sections were washed with 0.5% BSA in PBS. Then, the sections were incubated with TUNEL reaction mixture (Roche Diagnostic Gmbh, Penzberg, Germany) for 1 h at 37 °C. After washing in PBS, the sections were mounted using VECTASHIELD mounting medium with DAPI (Vector Laboratories, Burlingame, CA, USA) and visualized with a confocal microscope (LSM710, Carl Zeiss, Jena, Germany).

### Cell culture

Immortalized organ of Corti cells derived from mice^34^, the University of Bristol organ of Corti (UB/OC1) cells were cultured in high-glucose DMEM (Hyclone, Cramlington, UK) in the presence of γ-interferon with 10% fetal bovine serum (GIBCO, Grand Island, NY, USA) at 33 °C in an incubator containing 10% CO_2_. To induce differentiation, the cells were cultured at 37 °C without γ-interferon under 5% CO_2_.

### siRNA transfection

siRNAs were synthesized by Bioneer Inc. (Bioneer, Daejeon, Korea). The target sequence for siRNAs was as follows: 5ʹ-GAAAUGUCAACGUGUUCAA-3ʹ for LDHB and the nonspecific-negative control (siControl). The siRNAs were transfected into UB/OC1 cells using Thermo Scientific DharmaFECT1 transfection reagent (Thermo Fisher Scientific, Waltham, MA, USA).

### Cell viability assay

Cell viability was determined using the 3-(4,5-dimethyl-thiazol-2-yl)-2,5-dipheny-2H-tetrazolium bromide (MTT) assay (Promega, Southampton, UK). Auditory cells were treated with various concentrations of oxidative drugs and mitochondrial toxins for 24 h. At the end of incubation, MTT solution was added to each well. Absorbance was measured using an iMark™ Microplate Absorbance Reader (Bio-Rad, Hercules, CA, USA) at 595 nm. All assays were performed at least three times and viability was normalized to the control.

### Measurement of intracellular ATP

ATP was monitored through detection of light caused by the reaction of ATP with added luciferase and D-luciferin. Following the manufacturer’s instructions for the ATPlite™ Luminescence Assay System 1000 Assay Kit (PerkinElmer, Waltham, MA, USA), cells were lysed in ATPLite lysis buffer (ATPlite: NaOH, 100 Mriton X-100 4 ml/l) with shaking and sonication. The cell lysate was centrifuged at 13,000 rpm for 5 min at 4 °C, and the supernatant was measured. In a 96-well microplate, 65 μl of dilution buffer (PBS:lysis buffer = 2:0) was mixed with 10 μl of sample and 15 μl of substrate solution, kept in dark conditions for 10 min, and the luminescence intensity was measured. The ATP level was normalized to the protein concentration.

### Measurement of MMP

The MMP of intact cells was determined through flow cytometry with tetramethylrhodamine methyl ester (TMRM; Molecular Probes, Life Technologies), which is a potentiometric, cell permeable fluorescent indicator that accumulates in the highly negatively charged interior of mitochondria. The culture medium was removed from adherent UB/OC1 cells, rinsed with PBS, and re-suspended in 500 μl DMEM with 5 μl of 1 mM TMRM (10 μM final concentration), and incubated at 37 °C for 30 min. The cells were treated with CCCP for 10 min prior to TMRM staining. Fluorescence was quantified using flow cytometry (FACS Vantage, Becton Dickinson).

### Measurement of ROS

Intracellular ROS level was monitored through flow cytometry with the fluorescent probe 2ʹ,7ʹ-dichlorodihydrofluorescein (CM-H_2_DCFDA; Molecular Probes). Cells were rinsed with PBS prior to pelleting and re-suspended in 500 μl PBS with 5 μM CM-H_2_DCFDA, then incubated at 37 °C for 30 min. Fluorescence was quantified using flow cytometry (FACS Vantage, Becton Dickinson).

### NAD^+^/NADH ratio assay

Whole-cell NAD^+^ and NADH were measured using the Elite Fluorimetric NAD/NADH ratio assay kit (eENZYME, LLC, Gaithersburg, MD, USA) according to the manufacturer’s instructions. Briefly, cells were lysed with NAD^+^/NADH lysis buffer using a freeze-thaw cycle and sonication. The lysate was centrifuged at 13,000 rpm and 4 °C for 15 min and the supernatant was collected as a sample. NAD^+^, NADH, and NAD^+^/NADH ratio measurements were based on an enzymatic cycling reaction, measured using a fluorescent plate reader (Ex/Em = 540/590). The NADH level was normalized to protein concentration.

### LDH activity

Enzyme activity was determined by measuring the change in absorbance at 340 nm, as previously described^23^. The cells were lysed in lysis buffer (100 mM K_2_HPO_4_, 30 mM KF, 1 mM EDTA, and protease inhibitor cocktail) with homogenization. The lysate was centrifuged at 13,000 rpm and 4 °C for 15 min, then the supernatant was collected as a sample. Spectrophotometric assays of pyruvate to lactate (P-L) were performed at 25 °C and pH 7.4 (0.1 M potassium phosphate buffer), with a final NADH concentration of 0.25 mM, and 0.5 mM sodium pyruvate. The assay of lactate to pyruvate (L-P) LDH activity was performed at 25 °C and pH 8.8 (50 mM sodium pyrophosphate buffer), with final concentrations of 5.25 mM NAD and 5 mM lactate. In all assays, absorbance at 340 nm was measured at 1 min intervals for 10 min with a FlexStation microplate reader. We used Vmax as the main parameter of LDH activity, normalized to protein concentration.

### Lactate production assay

The lactate level in culture media was analyzed using a spectrophotometric assay, as described in a previous study^35^. Briefly, 100 μl of culture medium was mixed with 200 μl perchloric acid and centrifuged at 3000 g for 15 min; the supernatant was neutralized with potassium hydroxide (KOH) (3 M) at a ratio of 400:17, then incubated on ice for 15 min and centrifuged at 3000 × *g* for 15 min. The supernatant was applied to the spectrometric lactate assay using a Thermo Max microplate reader (Molecular Devices Co., Sunnyvale, CA, USA). The lactate level was assessed using a standard lactate calibration curve prepared under the same conditions and expressed as lactate (μg) released from 1 μg of cell lysate.

### LDH isozyme pattern assay

The LDH isozyme pattern was identified with non-denaturing Tris-glycine PAGE. Proteins were lysed in lysis buffer (100 mM K_2_HPO_4_, 30 mM KF, 1 mM EDTA, and protease inhibitor cocktail) with homogenization, the lysate was centrifuged at 13,000 rpm and 4 °C for 15 min, and the supernatant was used as a sample. Samples were kept on ice at all times. Loading samples were prepared by adding 40% sucrose with the same volume as a sample, colored with bromophenol blue, and 10-μg samples were loaded in each well and separated using SDS-free Tris-glycine electrophoresis buffer. Then, the gel was stained with developer solution: lactate (3.24 mg/ml), β-nicotinamide adenine dinucleotide (NAD^+^; 0.3 mg/ml), nitroblue tetrazolium (NBT; 0.8 mg/ml), and phenazine methosulfate (PMS; 0.167 mg/ml) dissolved in 10 mM Tris-HCl (pH 8.5) buffer. The gel was incubated at 37 °C for 30 min or longer, until the expected bands were seen, and then finished through reaction with 5% acetic acid.

### Western blot assay

Total proteins were extracted using radioimmunoprecipitation assay buffer. Protein concentration was assessed with a Bio-Rad DC protein assay (Bio-Rad Laboratories, Hercules, CA, USA). Proteins were separated by electrophoresis on sodium dodecyl sulfate polyacrylamide gels. The same amount of protein (μg) was loaded in each lane. After electrophoresis, the proteins were transferred to a polyvinylidene difluoride or nitrocellulose membrane and subsequently subjected to immunoblotting analysis using appropriate antibodies. The amount of loading was further determined using a western blotting housekeeping protein (lamin A, 1:5000 dilution). Using a horseradish peroxidase-conjugated secondary antibody, protein bands on the blots were visualized with enhanced chemiluminescent western blot detection reagent. Antibodies including anti-tubulin (Abcam, ab7291), anti-LDHB (Abcam, ab75167), anti-complex I (Abcam, ab110242), anti-complex II (Abcam, ab14714), anti-complex III (Abcam, ab14745), anti-complex IV (Abcam, ab14705), anti-complex V (Abcam, ab14748) were purchased from commercial company (Abcam, Cambridge, UK).

### Statistical analysis

Data are expressed as mean ± standard deviation. Significant differences between two independent groups were analyzed with Student’s *t*-test using IBM SPSS Statistics for Windows (version 21.0, Amonk, NY, USA), and *P*-values ≤ 0.05 were considered significant. When we performed *t*-test, if the two samples were satisfied with the assumption of equal variance, we used *t*-test for equal variances, whereas we applied *t*-test for unequal variances if the samples’ values showed unequal variances.

## Supplementary information

Supplementary Figure 1

Supplementary Figure 2

Supplementary Figure Legends
