# Supplementary figures and images for: Suppressed expression of LDHB promotes age-related hearing loss via aerobic glycolysis
Source: Cell Death Dis. 2020 May 15;11(5):375. doi: 10.1038/s41419-020-2577-y (PMC7229204; doi:10.1038/s41419-020-2577-y)

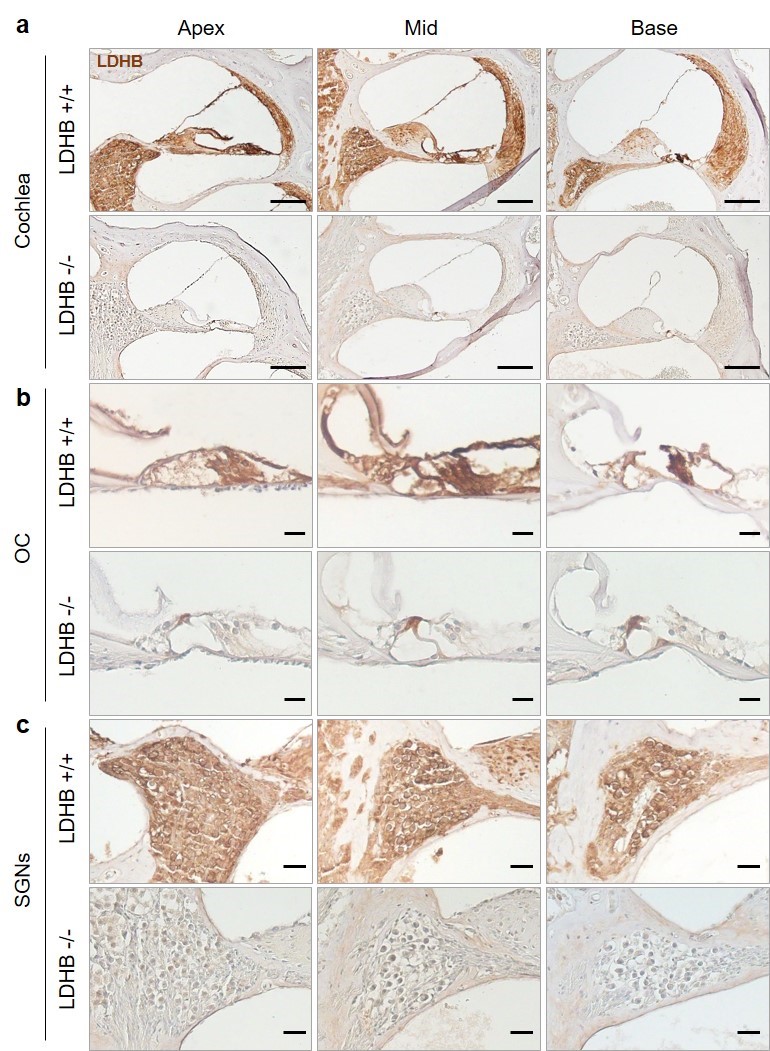

Supplement: Supplementary file 1 — Supplementary Figure 1 [file 41419_2020_2577_MOESM1_ESM.jpg]

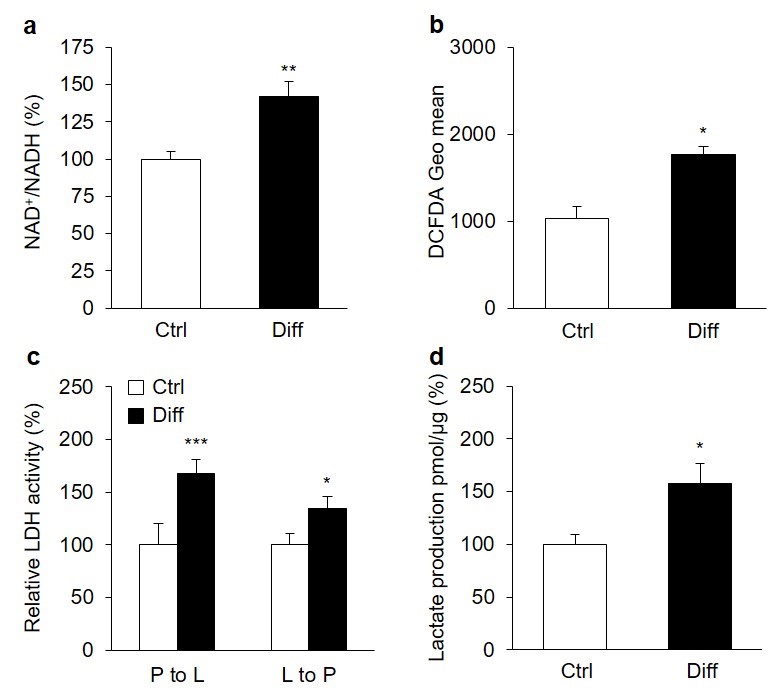

Supplement: Supplementary file 2 — Supplementary Figure 2 [file 41419_2020_2577_MOESM2_ESM.jpg]
