# Supplementary material for: Suppressed expression of LDHB promotes age-related hearing loss via aerobic glycolysis
Source: Cell Death Dis. 2020 May 15;11(5):375. doi: 10.1038/s41419-020-2577-y (PMC7229204; doi:10.1038/s41419-020-2577-y)
Supplement: Supplementary file 3 — Supplementary Figure Legends [file 41419_2020_2577_MOESM3_ESM.docx]

**Suppressed expression of LDHB promotes age-related hearing loss via aerobic glycolysis**

Chunjie Tian^1,*^, Yeon Ju Kim^2,*^, Sai Hali^3,*^, Oak-Sung Choo^2,4^, Jin-Sol Lee^2,5^,

Seo-Kyung Jung,^2,5^, Youn-Uk Choi^6^, Chan Bae Park^6^ and Yun-Hoon Choung^2,4,5^

^1^ Department of Otolaryngology, Dali Bai Autonomous Prefecture People's Hospital, Dali, 671000, Yunnan, China

^2^ Department of Otolaryngology, Ajou University School of Medicine, Suwon, 16499, Republic of Korea

^3^ Institute for Medical Sciences, Ajou University School of Medicine, Suwon, 16499, Republic of Korea

^4^ Department of Medical Sciences, Ajou University Graduate School of Medicine, Suwon, 16499, Republic of Korea

^5^ Department of Biomedical Sciences, BK21 Plus Research Center for Biomedical Sciences, Ajou University Graduate School of Medicine, Suwon, 16499, Republic of Korea

^6^ Department of physiology, Ajou university school of medicine, Suwon, 16499, Republic of Korea

^*^First three contributed equally to this work

Co-Correspondence to:

Yun-Hoon Choung, MD, DDS, PhD

Department of Otolaryngology, Ajou University School of Medicine, San 5, Woncheon-dong, Yeongtong-gu, Suwon 443-721, Republic of Korea.

Tel: +82-31-219-5263; Fax: +82-31-219-5264; E-mail address: [yhc@ajou.ac.kr](mailto:yhc@ajou.ac.kr)

ORCID: 0000-0002-0786-1781

Chan Bae Park, PhD

Department of Physiology, Ajou University School of Medicine, San 5, Woncheon-dong, Yeongtong-gu, Suwon 443-721, Republic of Korea.

Tel: +82-31-219-4560; Fax: +82-31-219-4630; E-mail address: pcbkaist@gmail.com

**Running title**

The role of LDHB in age-related hearing loss

**Supplementary Figure Legends**

**Supplementary Fig. 1.** Characterization of LDHB KO mice. Immunohistochemistry of LDHB (brown, DAB) in WT and LDHB KO mice at 5 months of age. Cochlear sections were counterstained with hematoxylin (blue). Scale bars: 100 µm (a) and 20 µm (b, c). OC, organ of Corti; SGNs, Spiral ganglion neurons

**Supplementary Fig. 2** LDHB levels and mitochondrial functions of differentiated UB/OC1 cells. **a-d** UB/OC1 cells were cultured under permissive conditions of 37^o^C and 5% CO_2_ without γ-interferon to allow differentiation into hair cells. The ratio of NAD^+^/NADH (**a**), ROS levels as measured by DCFDA fluorescence (**b**), LDH activity (**c**), and lactate level (**d**) in differentiated UB/OC1 cells were compared to those of the control cells. White bars represent undifferentiated control cells (Ctrl) and black bars represent differentiated cells (Diff). **P* < 0.05, ***P* < 0.01, ****P* < 0.001 vs undifferentiated cell by Student’s t-test.
